# The intergenerational transmission of language skill

**DOI:** 10.1111/1468-4446.12780

**Published:** 2021-02-17

**Authors:** Alice Sullivan, Vanessa Moulton, Emla Fitzsimons

**Affiliations:** ^1^ Department of Social Science Centre for Longitudinal Studies (CLS) UCL Institute of Education London UK

**Keywords:** education, ethnicity, inequality, intergenerational, language, vocabulary

## Abstract

This paper examines the relationship between parents’ and children's language skills for a nationally representative birth cohort born in the United Kingdom—the Millennium Cohort Study (MCS). We investigate both socioeconomic and ethnic differentials in children's vocabulary scores and the role of differences in parents’ vocabulary scores in accounting for these. We find large vocabulary gaps between highly educated and less educated parents, and between ethnic groups. Nevertheless, socioeconomic and ethnic gaps in vocabulary scores are far wider among the parents than among their children. Parental vocabulary is a powerful mediator of inequalities in offspring's vocabulary scores at age 14, and also a powerful driver of change in language skills between the ages of five and 14. Once we account for parental vocabulary, no ethnic minority group of young people has a negative “vocabulary gap” compared to whites.

## 1 INTRODUCTION

Social class differences in language use have been central to some of the major sociological theories regarding the intergenerational transmission of educational advantage. For Bourdieu, “linguistic competence” is central to educational and social reproduction (Bourdieu, 1977; Bourdieu, Passeron, & Saint‐Martin, 1994). Despite this, measures of linguistic attainment have rarely been used in empirical operationalizations of Bourdieu's theory of cultural reproduction (Sullivan, 2001). Bernstein also placed substantial weight on the differences in language use by middle and working‐class children, arguing that this affected their ability to succeed at school (Bernstein, 1971, 1973, 1975). Language knowledge is clearly an important prerequisite for school learning, and language difficulties have been linked to a range of adverse outcomes (Law, Rush, Schoon, & Parsons, 2009). However, most empirical studies have neglected the role of parental language skills. This means we do not know to what extent parental language skills are transmitted to the child, or how important parental language skills are in explaining socioeconomic gaps in children's language skills.

Perhaps the most widely cited empirical source on the question of the relationship between parents’ and children's vocabularies is Hart and Risley’s (1995) observational study. Hart and Risley's research on 42 families in one U.S. college town found strong social class and black‐white differences in the range of vocabulary used by parents when talking to their children. Their headline finding that “professional class” children had been exposed to 30 million more words than “welfare children” had by age three (Hart & Risley, 2003) has been enormously influential, despite the drawback of a small and unrepresentative sample. (To be clear, the 30 million figure does not refer to unique words, but the total barrage of speech to which the children were exposed, including repetition). The study is contested (Golinkoff, Hoff, Rowe, Tamis‐LeMonda, & Hirsh‐Pasek, 2018; Sperry, Sperry, & Miller, 2018), but important, given the lack of evidence in this field. We are aware of only one previous large quantitative study which assesses the role of parental vocabulary in shaping class and race differences in children's vocabularies (Farkas & Beron, 2004).

### 1.1 The current study

We exploit unique new data on the vocabulary scores of both parents and children in a nationally representative UK birth cohort study. A distinctive feature of the study is that mothers, partners, and children took an equivalent vocabulary test when the children were aged 14. This allows us to build on the existing evidence base in a number of important ways. First of all, we are able to establish the vocabulary gaps that exist for both parents and children according to social class and ethnic group using a nationally representative birth cohort study. Second, we address the extent to which socioeconomic and ethnic gaps in children's scores at age 14 are driven by differences in the parents’ scores. Third, we assess the extent to which the role of the home environment and the child's own cultural capital are reduced once parental vocabulary is taken into account. Finally, given that language scores on school entry are a strong predictor of later language acquisition (Duncan et al., 2007), we assess whether parental vocabulary is associated with a growing language gap for children between the ages of five and 14.

## 2 BACKGROUND

### 2.1 The development of language gaps

Socioeconomic differentials in both verbal and general cognitive attainment emerge early in life, and widen during the pre‐school and school years (Becker, 2011; Byford, Kuh, & Richards, 2011; Chiu & Chow, 2010; Douglas, 1964; Farkas & Beron, 2004; Feinstein, 2003; Fogelman & Goldstein, 1976; Law, McBean, & Rush, 2011; Law, Rush, King, Westrupp, & Reilly, 2017; Layte, 2017; Ready, 2010; Sullivan & Brown, 2015a; Sullivan, Ketende, & Joshi, 2013). The vast majority of the literature examining the relationship between parental and child vocabulary is focused on the early years, possibly because it is during this period that the greatest challenges are met (Cartmill et al., 2013; Fernald, Marchman, & Weisleder, 2013; Rowe, 2012). Yet social class differentials in vocabulary continue to grow during adolescence and even into mid‐life (Sullivan & Brown, 2015a, 2015b).

### 2.2 What explains socioeconomic language gaps?

From the perspective of cultural reproduction theory, the ability to understand and use “educated” language is a vital part of the advantage that is transmitted by high‐status parents (Bourdieu, 1977). In this sense, language can be seen as part of the cultural capital that is transmitted within the home. But there is an ambiguity within Bourdieu's work regarding the role of language—is language a fundamental building block of learning, or is it simply a signal of class membership, which is arbitrarily rewarded by the education system? Of course, these are not mutually exclusive. It may be the case both that building vocabulary is vital for learning across subjects, and that educators sometimes arbitrarily reward styles of expression associated with elite groups. However, our focus is on language as a tool for communication and learning, and hence on quantitative differentials in vocabulary rather than on qualitative differences in styles of expression.

The concept of cultural capital has been operationalized in diverse ways. A useful distinction has been drawn between “status‐seeking” and “information processing” forms of cultural capital (Ganzeboom, 1982). Information processing cultural capital leads to the development of knowledge and skills which are rewarded in the education system (Sullivan, 2002). Status‐seeking cultural capital is rewarded via teacher bias rather than improved skills (Farkas, Grobe, Sheehan, & Shuan, 1990; Jæger & Møllegaard, 2017). We prefer the terms “literary cultural capital” and “non‐literary” cultural capital to refer to cultural activities that relate to books and reading and those that do not. Studies that have separated the two have found that literary cultural capital has more influence on educational attainment (De Graaf, De Graaf, & Kraaykamp, 2000; Jæger, 2011; Sullivan, 2001).

Psychologists have also stressed the importance of the home literacy environment to children's language learning (Melhuish et al., 2008; Waldfogel & Washbrook, 2011). Three aspects of parenting have been highlighted as central to children's early language and learning (Rodriguez et al., 2009): (a) frequency of children's participation in routine *learning activities* (e.g., shared book reading, storytelling); (b) the *quality of caregiver‐child engagements* (e.g., parents’ cognitive stimulation and sensitivity/responsiveness); and (c) the provision of age‐appropriate *learning materials* (e.g., books and toys). Studies have found substantial socioeconomic differentials in these parental inputs (Bassok, Finch, Lee, Reardon, & Waldfogel, 2016). However, studies assessing the role of the home literacy environment have not accounted for the role of parental language skills. This is important because it is likely both that parents who have strong language skills will be most comfortable engaging in activities such as shared reading, and also that they may be more effective at engaging their children in these activities than, for example, a parent who struggles with basic literacy skills (Sullivan et al., 2013). Evidence suggests that assortative mating according to verbal cognitive scores is notably higher than for non‐verbal scores (Plomin & Deary, 2015).

Regardless of the theoretical perspective applied, empirical findings across the disciplines highlight the importance of the home literary climate. Whether books in the home are termed “embodied cultural capital” or “learning materials,” they remain a powerful predictor of educational outcomes (Marks, Cresswell, & Ainley, 2006). Both theoretical perspectives have merit, as books in the home are learning materials, but also reflect the value placed upon books and learning within the family, and represent a cultural display, signaling that the owner is a cultured person. Parental reading to children (Bus, Van Ijzendoorn, & Pellegrini, 1995) and children's own reading (Stanovich & Cunningham, 1998; Sullivan & Brown, 2015a) are powerful predictors of both language learning and wider educational outcomes.

### 2.3 Ethnic differentials

Findings regarding ethnic gaps in both cognitive and educational attainment vary widely according to the minority groups considered and the particular national context (Alba & Waters, 2011). Much of the sociological literature relates to the U.S. context, where black‐white test score gaps form in early childhood and widen during the school years (Jencks & Phillips, 1998; Quinn, 2015b). It is, therefore, important to be clear about the important differences between the US and UK contexts. Waters et al. (2013) provide a useful overview of the main immigrant groups and differences in the United Kingdom and United States experience. Key points of difference are that black people in the United Kingdom, especially the black Caribbean group, have high levels of intermarriage and residential integration with whites compared to black people in the United States. In contrast, while Pakistani and Bangladeshi immigration to the United Kingdom began in the 1960s and 1970s, the widespread practice of transnational arranged cousin marriage maintains an ongoing “first generation” for many families, with associated lower tendency to speak English at home (Sullivan, 2010). In the United Kingdom, children of Pakistani and Bangladeshi ethnic backgrounds start at a disadvantage, but make greater progress during schooling than whites (Hoffmann, 2018; Strand, 2011; Sullivan et al., 2013), and white and black Caribbean working‐class students are generally the lowest achievers at school (Strand, 2014). UK ethnic minorities as a whole are more likely than whites to gain a university degree (Modood, 2004), and all ethnic minority groups in the United Kingdom are more likely to enter university than their white peers with similar prior attainment (Belsky, Barnes, & Melhuish, 2007). Theories regarding social class differences in the transmission of educational advantages and disadvantages cannot simply be mapped onto ethnic differences. Particularly in the case of immigrant groups, the educational fates of children may not be as strongly tied to the parents’ current status as class‐based theories would lead us to predict.

## 3 RESEARCH QUESTIONS

While researchers from a range of theoretical and disciplinary perspectives have emphasized the importance of inequalities in language development, a number of important empirical questions remain unanswered.

1. How large are vocabulary gaps according to childhood socioeconomic circumstances, ethnic group, and other factors?
2. What is the role of the home literacy culture in predicting child vocabulary and explaining SES gaps? We hypothesize that the come literacy culture predicts child vocabulary, net of socioeconomic background.
3. What is the role of the child's own cultural capital in predicting vocabulary and explaining SES gaps? We assess the roles of reading for pleasure (literary cultural capital) and playing an instrument (non‐literary cultural capital). We hypothesise that reading for pleasure is an important predictor of vocabulary, whereas playing an instrument is less important.
4. How important are the mother's and partner's vocabulary in predicting the child's vocabulary, and does this substantially mediate SES and other differentials in the model? We hypothesise that parental vocabulary is of primary importance as a predictor of offspring's vocabulary, and mediates SES differences.
5. Which factors are relevant for progress in verbal scores between the ages of five and 14? We hypothesise that parental vocabulary, the home literary culture, and the child's own reading are predictors of vocabulary scores at 14, net of earlier vocabulary scores

## 4 DATA AND MEASURES

The Millennium Cohort Study (MCS) is a national birth cohort study following the lives of 19,517 children born in the United Kingdom in 2000–2001 (Connelly & Platt, 2014; Joshi & Fitzsimons, 2016). The sample comprised all those born within eligible dates (September 1, 2000–August 31, 2001 in England and Wales; November 23, 2000–January 11, 2002 in Scotland and Northern Ireland), and resident in a stratified sample of electoral wards. The initial response rate was 72% of all families with eligible children living at nine months in the sampled wards (Plewis, Calderwood, Hawkes, Hughes, & Joshi, 2007). There have been six waves of data collection, at ages 9 months and 3, 5, 7, 11, and 14 years. The seventh, age 17 wave, is in the field at the time of writing. The study is multi‐disciplinary and contains rich repeat measures of childhood socioeconomic circumstances, child development, and child health. The MCS datasets are freely available to researchers internationally via the UK Data Service (http://ukdataservice.ac.uk). The CLS website provides detailed information and documentation on the study (http://www.cls.ioe.ac.uk/mcs).

Eleven thousand seven hundred fourteen households responded at the sixth wave of data collection (MCS6). This represents a response rate of 76% of those issued to the field at sweep 6 and just under 61% of the initial sample. Of these, cohort members in 10,781 households completed the vocabulary test. This group is our analytical sample. We use records for only one child per family (singletons and the first‐born twin or triplets) to avoid having to account for the clustering of children within families. We exploit data provided by the “main respondent” parent (this is typically the mother, and we refer henceforth in the text to mothers rather than main respondents), the spouse or cohabiting partner (where applicable), and the child themselves, up to age 14. More information on data collection and attrition is available in the MCS6 technical report (Ipsos Mori, 2017).

We exploit data from birth to age 14, and, as in any longitudinal analysis, the problem of missing data must be addressed (Mostafa & Wiggins, 2015). It is well known that list‐wise deletion/complete case analysis returns biased estimates, so we use multiple imputation with chained equations (25 imputed datasets) to “fill‐in” values of any missing items in the variables selected for our analysis adopting Schafer's data augmentation approach (Schafer, 1997) under the assumption of “missing at random” (MAR). In order to maximise the plausibility of the MAR assumption we also include a set of auxiliary variables in our imputation model. In this instance, MAR implies that our estimates are valid if missingness is due to variables (auxiliary or substantive) that were included in our models (Little & Rubin, 2002). In addition, to take account of disproportionate, stratified clustering in the MCS sample design and attrition, models are adjusted for non‐response and the MCS survey design. The combination of multiple imputation and non‐response weighting restores the sample to be nationally representative of the UK population born in 2000–2001 (Fitzsimons, 2017). We impute the full sample, but delete cases for which the outcome is missing (Hippel & Paul, 2007).

### 4.1 Measures

#### 4.1.1 Language skill

The mother, partner, and child's vocabulary scores were assessed when the cohort member was aged 14. Vocabulary is strongly associated with other dimensions of verbal ability (Baddeley, Logie, Nimmo‐Smith, & Brereton, 1985). The vocabulary scores were derived from a shortened version of the Applied Psychology Unit (APU) Vocabulary Test, a standardised test produced by the University of Edinburgh (Closs & Hutchings, 1976), and used in previous studies including the 1970 British Cohort Study (BCS70). The APU Vocabulary Test directly examines vocabulary knowledge, through multiple‐choice items in which a stimulus word has to be matched to a synonym from five alternatives. At the start of the test the stimulus words are very easy, for example, “begin” and become progressively more difficult; for example, “pusillanimous,” The final score is the sum of the correct answers, from a total of 20 multiple‐choice items. The APU test has previously been shown to have good psychometric properties and is highly correlated with other tests of verbal intelligence (Levy & Goldstein, 1984). We provide information on the internal reliability and distribution of the vocabulary scores for each respondent in Appendix. Although the test was developed for teenagers, within our sample, the internal reliability scores are higher for the adult respondents.

#### 4.1.2 Socioeconomic and demographic factors

The socioeconomic and demographic information in our models includes the age (in months), sex and ethnic group of the child, and the region of the United Kingdom that the family lives in. Parents’ education is the highest qualification of either parent. Economic circumstances are captured in wave 1 of the survey in 2001–2002 (or wave 2 in 2004–2005 if not available at wave 1) by parental social class measured on the National Statistics SocioEconomic Classification (NS‐SEC) scale (Goldthorpe & McKnight, 2006), home ownership, and log equivalised family income. The number of older and younger siblings is included, as older siblings have been shown to be advantaged both in terms of vocabulary and general cognitive outcomes (Black, Devereux, & Salvanes, 2005; Hoff‐Ginsberg, 1998; Nisbet, 1953). Whether English is the main language spoken at home at wave 1 of the survey (or wave 2 where unavailable at wave 1) is included, as this may be related to both parental and child vocabulary scores. We include the ages of the mother, partner, and child in the model, as vocabulary is expected to increase with age, especially in the case of the child. In addition, the models control for single‐parent household status at wave 6 (in 2015), and, if the mother's partner was present, whether they completed the vocabulary test or not.

#### 4.1.3 Home literary climate

Books in the home and the frequency of parental reading to the child at age three.

#### 4.1.4 Child's cultural capital

The child's own reading frequency and playing a musical instrument at age 11 (self‐reported).

#### 4.1.5 Early cognitive scores

Cognitive abilities at age five were measured using three subscales of the British Ability Scales Second Edition (BAS II): naming vocabulary, picture similarities, and pattern construction. The three subscales capture core aspects of verbal and pictorial reasoning, and spatial abilities (Elliott, Murray, & Pearson, 1978; Elliott, Smith, & McCulloch, 1996; Hill, 2005; Jones & Schoon, 2008).

## 5 RESULTS

We begin by describing mean parental and child vocabulary scores according to the other variables to be used in our models and assessing the correlations between cognitive measures. This is followed by a series of linear regression models, with child vocabulary at age 14 as the outcome. Finally, we present a path analysis as a formal test of the mediation of the effect of parental education on child vocabulary by parental vocabulary and other factors.

### 5.1 Descriptive results

Table 1 presents raw (imputed and weighted) mean scores out of 20 in the vocabulary assessment, by respondent type (young person, mother, and partner). Young people achieved a mean score of seven out of 20 on average, while mothers and partners gained substantially higher scores (10 and 11, respectively). The standard deviation is also higher for the parents than for the child, reflecting a wider spread of scores.

**TABLE 1:** Mean vocabulary scores for young people, mothers, and partners (imputed and weighted)

	Sample %	Original *N*	% missing	Young person's vocabulary (mean and 95% CI)	Main vocabulary (mean and 95% CI)	Partner vocabulary (mean and 95% CI)
	*n* = 10,781			*n* = 10,781	*n* = 10,781	*n* = 8,178
Overall				6.88 (6.78, 6.97) *SD* = 2.58	10.32 (10.09, 10.54) *SD* = 4.32	11.25 (10.97, 11.52) *SD* = 4.27
Age (months) quartile		10,781				
1st	27.8			6.81 (6.67, 6.94)	10.40 (10.11, 10.68)	11.16 (10.81, 11.50)
2nd	21.2			6.88 (6.72, 7.04)	10.45 (10.15, 10.74)	11.42 (11.07, 11.76)
3rd	24.0.9			6.91 (6.76, 7.06)	10.37 (10.07, 10.68)	11.44 (11.07, 11.81)
4th	26.1			6.92 (6.79, 7.04)	10.07 (9.78, 10.36)	11.02 (10.65, 11.39)
Gender		10,781				
Male	51.9			6.86 (6.74, 6.98)	10.33 (10.09, 10.57)	11.19 (10.91, 11.47)
Female	48.1			6.90 (6.79, 7.01)	10.30 (10.04, 10.57)	11.31 (10.99, 11.63)
Ethnicity		10,781				
White	80.4			6.96 (6.87, 7.06)	11.00 (10.80, 11.20)	11.99 (11.78, 12.20)
Mixed	4.9			6.94 (6.62, 7.26)	10.58 (10.02, 11.15)	12.25 (11.49, 13.01)
Indian	2.1			6.60 (5.95, 7.24)	7.44 (6.27, 8.60)	8.55 (7.55, 9.54)
Pakistani	3.7			6.16 (5.90, 6.43)	5.88 (5.39, 6.36)	5.87 (5.18, 6.56)
Bangladeshi	1.4			6.47 (6.15, 6.79)	4.53 (3.73, 5.33)	4.36 (3.61, 5.11)
Black Caribbean	1.5			6.44 (5.86, 7.03)	7.96 (7.05, 8.86)	10.52 (8.59, 12.46)
Black African	2.4			6.50 (6.20, 6.80)	6.77 (5.88, 7.65)	7.67 (6.05, 9.27)
Other ethnic group	3.6			6.44 (6.10, 6.78)	6.53 (5.83, 7.24)	6.60 (5.72, 7.49)
Country		10,781				
England	71.2			6.89 (6.80, 6.99)	10.51 (10.21, 10.82)	11.37 (10.99, 11.74)
Scotland	8.4			6.84 (6.64, 7.03)	10.95 (10.56, 11.34)	12.21 (11.71, 12.70)
Wales	5.0			6.62 (6.44, 6.80)	10.22 (9.88, 10.56)	10.97 (10.59, 11.35)
Northern Ireland	4.3			6.83 (6.67, 7.00)	9.96 (9.51, 10.42)	11.02 (10.50, 11.55)
London	11.1			7.01 (6.74, 7.28)	8.75 (7.89, 9.61)	9.79 (8.76, 10.82)
Household education		10,771	0.09			
No qualifications	12.0			5.84 (5.63, 6.06)	6.52 (6.06, 6.99)	7.03 (6.19, 7.87)
Vocational only	3.1			6.04 (5.68, 6.39)	7.39 (6.82 7.96)	8.60 (7.70, 9.49)
Other academic	2.2			6.34 (6.02, 6.66)	6.44 (5.80, 7.08)	6.29 (5.28, 7.31)
GCSEd‐g	9.4			6.15 (5.92, 6.39)	8.07 (7.78, 8.38)	9.22 (8.72, 9.72)
GCSEa‐c	34.2			6.57 (6.47, 6.68)	9.83 (9.64, 10.03)	10.43 (10.19, 10.67)
A level or HE diploma	8.5			7.12 (6.93, 7.32)	11.59 (11.28, 11.90)	12.08 (11.71, 12.44)
Higher Diploma	10.7			7.19 (7.01, 7.36)	11.35 (11.11, 11.58)	12.03 (11.78, 12.30)
First Degree	14.4			8.17 (8.02, 8.31)	13.96 (13.67, 14.25)	14.22 (13.94, 14.51)
Higher Degree	5.6			8.64 (8.34, 8.94)	15.10 (14.71, 15.49)	15.48 (15.09, 15.88)
Household social class		10,334	4.11			
Routine	9.0			5.99 (5.71, 6.28)	7.04 (6.61, 7.46)	8.08 (7.26, 8.90)
Semi‐routine	15.0			6.26 (6.09, 6.43)	8.18 (7.83, 8.53)	8.68 (8.12, 9.24)
Lower superv./tech.	10.2			6.35 (6.19, 6.51)	8.51 (8.20, 8.81)	9.31 (8.85, 9.76)
Small empl./self‐empl.	8.6			6.36 (6.15, 6.58)	8.63 (8.24, 9.02)	8.88 (8.35, 9.42)
Intermediate	12.3			6.80 (6.61, 6.98)	10.42 (10.16, 10.68)	10.84 (10.52, 11.17)
Lower manag./prof.	26.8			7.20 (7.09, 7.31)	11.80 (11.60, 12.00)	12.30 (12.08, 12.52)
Higher manag./prof.	18.1			8.14 (7.96, 8.31)	13.88 (13.55, 14.21)	14.35 (14.35, 14.91)

	Sample %	Original *N*	% missing	Young person's vocabulary (mean and 95% CI)	Main vocabulary (mean and 95% CI)	Partner vocabulary (mean and 95% CI)
	*n* = 10,781			*n* = 10,781	*n* = 10,781	*n* = 8,178
Home owner		10,767	0.13			
Yes	55.5			7.30 (7.19, 7.48)	11.71 (11.43, 11.98)	12.25 (11.95, 12.54)
No	44.5			6.35 (6.23, 6.47)	8.58 (8.35, 8.80)	9.42 (9.06, 9.79)
Income (quartile)		10,732	0.45			
1st	30.3			6.13 (6.00, 6.27)	7.98 (7.68, 8.27)	8.58 (8.09, 9.06)
2nd	26.2			6.71 (6.58, 6.84)	9.46 (9.23, 9.69)	10.08 (9.75, 10.41)
3rd	22.8			7.18 (7.04, 7.31)	11.45 (11.24, 11.67)	12.03 (11.80, 12.26)
4th	20.6			7.69 (7.69, 8.03)	13.58 (13.26, 13.89)	14.09 (13.80, 14.38)
Main age (quartile)		10,779	0.02			
1st	27.3			6.19 (6.04, 6.33)	8.37 (8.13, 8.61)	9.55 (9.17, 9.93)
2nd	26.6			6.90 (6.79, 7.02)	10.00 (9.74, 10.27)	10.91 (10.57, 11.25)
3rd	23.4			7.26 (7.12, 7.41)	11.42 (11.14, 11.70)	12.07 (11.72, 12.42)
4th	22.7			7.29 (7.14, 7.43)	11.88 (11.52, 12.24)	12.50 (12.14, 12.85)
Partner age (quartile)		8,175				
1st	27.8			6.46 (6.31, 6.61)	9.01 (8.67, 9.34)	9.64 (9.26, 10.02)
2nd	21.8			7.09 (6.92, 7.25)	10.84 (10.44, 11.24)	11.47 (11.16, 11.78)
3rd	24.0			7.11 (6.88, 7.33)	10.92 (10.39, 11.46)	11.96 (11.60, 12.31)
4th	26.3			6.88 (6.68, 7.08)	10.45 (9.91, 11.00)	12.80 (12.29, 13.31)
Home language		10,773	0.07			
English only	87.0			6.91 (6.82, 7.01)	10.86 (10.67, 11.05)	11.92 (11.72, 12.013
Mixed or non‐English	13.0			6.65 (6.47, 6.84)	6.66 (6.14, 7.17)	7.22 (6.57, 7.88)
Books in home		10,096	6.35			
<10	15.2			6.04 (5.88, 6.21)	7.21 (6.87, 7.55)	8.02 (7.44, 8.61)
11 to 25	15.3			6.37 (6.19, 6.55)	8.33 (8.01, 8.66)	9.42 (8.99, 9.86)
26 to 100	32.4			6.66 (6.54, 6.77)	10.02 (9.78, 10.27)	10.83 (10.55, 11.11)
101 to 200	18.7			7.27 (7.13, 7.42)	11.81 (11.54, 12.08)	12.55 (12.25, 12.84)
201 to 500	13.1			7.90 (7.69, 8.11)	13.60 (13.23, 13.97)	13.87 (13.53, 14.20)
>500	5.3			8.53 (8.14, 8.92)	14.50 (13.94, 15.06)	15.01 (14.53, 15.49)
Frequency read to child (age 3)		9,872	8.43			
Not at all	3.5			5.55 (5.21, 5.89)	6.21 (5.55, 6.87)	6.61 (5.63, 7.59)
Less often	2.3			6.05 (5.64, 6.47)	7.12 (6.55, 7.70)	8.51 (7.37, 9.64)
Once or twice a month	3.2			6.18 (5.84, 6.52)	8.42 (7.80, 9.04)	9.58 (8.46, 10.70)
Once or twice a week	15.8			6.34 (6.17, 6.51)	8.68 (8.34, 9.02)	9.49 (9.03, 9.96)
Several times a week	19.1			6.74 (6.59, 6.90)	9.97 (9.69, 10.25)	10.91 (10.56, 11.26)
Every day	56.1			7.25 (7.13, 7.37)	11.42 (11.17, 11.67)	12.24 (11.97, 12.50)
YP reads (age 11)		10,060	6.69			
Never	7.6			5.94 (5.71, 6.18)	9.21 (8.82, 9.60)	10.02 (9.41, 10.63)
Less often	7.5			6.43 (6.20, 6.66)	9.87 (9.43, 10.30)	10.51 (9.91, 11.17)
At least once a month	10.5			6.58 (6.44, 6.68)	9.97 (9.62, 10.31)	10.71 (10.24, 11.17)
At least once a week	29.5			6.56 (6.44, 6.68)	9.93 (9.66, 10.20)	10.87 (10.55, 11.20)
Most days	44.8			7.41 (7.28, 7.54)	10.94 (10.64, 11.23)	11.91 (11.56, 12.27)
Plays musical instrument (age 11)		10,194	6.23			
Yes	38.4			7.36 (7.22, 7.50)	11.34 (11.01, 11.64)	12.06 (11.74, 12.39)
No	61.6			6.59 (6.50, 6.68)	9.70 (9.49, 9.92)	10.71 (10.42, 11.01)

Note

Missing means imputed.

We observe stark ethnic differences (based on the young person's ethnic identification) in adult vocabulary scores. The parents of white and ethnically mixed young people had the highest mean scores (between 10.6 and 12.2)—around two and a half times higher than the parents of ethnically Bangladeshi young people, who received the lowest mean scores (between 4.4 and 4.5). These differentials to some extent reflect the prevalence of first‐generation immigrants among each ethnic group, for example, over 90% of Bangladeshi mothers were not born in the United Kingdom (Sullivan, 2010). As such, we would emphasize that minority parents’ lower vocabulary scores are likely to reflect a lack of English fluency, rather than wider ability or attainment. The ethnic gaps in the young people's scores are far more modest, ranging between a mean score of 6.2 for Pakistanis and 7 for ethnically mixed and white young people. This means that ethnic minority youth (excepting the mixed group) tend to have vocabulary scores that are relatively close to those of their parents, and in the case of Bangladeshis and Pakistanis, they achieve higher average scores than their parents. Regional differences in adult vocabulary scores are also apparent, with those living in London scoring lowest, reflecting the city's ethnically diverse population.

There are strong gradients in parental vocabulary scores according to parental education, social class, and income. Among households where at least one partner had a higher university degree, mothers scored an average of 15 out of 20, compared to 6.5 for households where neither parent had any formal educational qualification. The education gaps are less marked for the offspring than for the parents. The children of university graduates scored 8.6 versus 5.8 for children in households with no qualifications. For families with no parental qualifications, the mean score for children (5.8) is only around one correct answer less than for mothers (6.5). Children of parents with a higher degree scored an average of 8.6 compared to 15. A similar pattern is observed for social class, household income, and home ownership—parental socioeconomic vocabulary gaps are larger than those for young people.

Not surprisingly, adult English vocabulary is considerably lower among those whose home language is mixed or non‐English, compared to English only (6.7 and 10.9, respectively for mothers), but the difference among young people is negligible (6.6 vs. 6.9).

Turning to indicators of cultural resources, we see that both parents’ and children's vocabulary scores are higher in households with higher levels of books at home and more frequent reading to the child. Young people who read frequently have relatively high mean vocabulary scores, whereas their parents’ scores are less strongly differentiated according to this measure. The vocabulary gap between young people who play a musical instrument and those who do not is small (7.4 vs. 6.6).

Table 2 shows a correlation matrix of the young person, mother and partner vocabulary scores, and the child's early cognitive scores. The mother and partner scores are highly correlated, at around 0.5. This is in line with previous estimates of assortative mating for verbal intelligence (Plomin & Deary, 2015). Correlations of around 0.3 are observed between the young person and mother/partner. We also see higher correlations between earlier verbal cognition and age 14 vocabulary (0.36) than between early measures of spatial and pictorial reasoning and later vocabulary (0.25 and 0.20, respectively).

**TABLE 2:** Correlation matrix: Young person's, main and partner's vocabulary and young person's early cognition

	Young person vocabulary	Main vocabulary	Main vocabulary^a^	Partner vocabulary^a^	Naming vocabulary (age 5)	Picture similarities (age 5)
Young person vocabulary						
Main vocabulary	0.35 (0.02)					
Main vocabulary^a^	0.35 (0.02)					
Partner vocabulary^a^	0.32 (0.02)	0.43 (0.01)	0.53 (0.02)			
Naming vocabulary age 5	0.36 (0.01)	0.41 (0.02)	0.42 (0.02)	0.40 (0.02)		
Picture similarities age 5	0.20 (0.01)	0.19 (0.01)	0.19 (0.02)	0.18 (0.02)	0.34 (0.02)	
Pattern construction age 5	0.25 (0.01)	0.23 (0.01)	0.23 (0.02)	0.21 (0.02)	0.35 (0.02)	0.36 (0.01)
Observations	10,781	10,781	8,178	8,178	10,781	10,781

Note

Mean (*SE*). Missing observations are imputed.

^a^ Correlations for main and partner vocabulary where the partner is present in the household at MCS6 (*n* = 8,831).

### 5.2 Regression results

Table 3 shows a series of models predicting vocabulary scores at age 14. The outcome variable, parental vocabulary scores, and the child's prior cognitive scores are all standardized *z*‐scores.

**TABLE 3:** Vocabulary at age 14 (standardized): Linear regression

	Model 1: Demographics		Model 2: Family cultural capital		Model 3: Child cultural capital		Model 4: Parental vocabulary		Model 5: Child cognition	
	*B*	*SE*	*B*	*SE*	*B*	*SE*	*B*	*SE*	*B*	*SE*
Age (months)	0.01***	(0.00)	0.01***	(0.00)	0.01***	(0.00)	0.01***	(0.00)	0.00	(0.00)
Sex (ref: boys)	0.01	(0.02)	0.01	(0.02)	−0.05*	(0.02)	−0.04*	(0.02)	−0.06**	(0.02)
Ethnicity (ref: White)										
Mixed	−0.02	(0.06)	−0.02	(0.06)	−0.00	(0.06)	−0.02	(0.05)	−0.04	(0.05)
Indian	−0.21*	(0.09)	−0.14	(0.09)	−0.13	(0.09)	0.02	(0.09)	0.02	(0.08)
Pakistani	−0.18**	(0.06)	−0.11+	(0.06)	−0.11+	(0.06)	0.19*	(0.09)	0.18**	(0.06)
Bangladeshi	−0.12	(0.09)	−0.04	(0.09)	−0.06	(0.09)	0.19*	(0.09)	0.29***	(0.08)
Black Caribbean	−0.33***	(0.10)	−0.29	(0.10)	−0.31**	(0.10)	−0.19+	(0.11)	−0.13	(0.11)
Black African	−0.23**	(0.08)	−0.16*	(0.07)	−0.16*	(0.08)	0.01	(0.08)	0.07	(0.08)
Other Ethnic Group	−0.25***	(0.07)	−0.17*	(0.07)	−0.20**	(0.07)	−0.02	(0.07)	0.07	(0.07)
Country (ref: England)										
Scotland	−0.08*	(0.03)	−0.06+	(0.03)	−0.05	(0.03)	−0.06*	(0.03)	−0.06*	(0.03)
Wales	−0.11**	(0.03)	−0.10***	(0.03)	−0.09**	(0.03)	−0.07*	(0.03)	−0.08**	(0.03)
Northern Ireland	0.00	(0.04)	0.02	(0.03)	0.01	(0.03)	0.04	(0.04)	−0.00	(0.04)
London	0.11*	(0.05)	0.10*	(0.04)	0.09*	(0.04)	0.10*	(0.04)	0.11*	(0.04)
Partner present (ref: completed vocab)										
Partner present vocab missing	−0.04	(0.03)	−0.03	(0.03)	−0.03	(0.03)	−0.02	(0.03)	−0.00	(0.03)
Lone parent	−0.06	(0.05)	−0.02	(0.05)	−0.00	(0.05)	−0.09	(0.13)	−0.05	(0.11)
Highest Household academic qualification (ref: None)										
Vocational only	0.05	(0.08)	0.03	(0.08)	0.03	(0.07)	0.03	(0.07)	0.02	(0.08)
Other academic	0.16+	(0.09)	0.12	(0.09)	0.11	(0.09)	0.08	(0.09)	0.06	(0.08)
GCSEd‐g	0.07	(0.05)	0.05	(0.05)	0.06	(0.05)	0.00	(0.05)	−0.03	(0.05)
GCSEa‐c	0.17***	(0.04)	0.11**	(0.04)	0.11**	(0.04)	0.01	(0.04)	−0.04	(0.04)
A level or HE diploma	0.31***	(0.06)	0.21***	(0.06)	0.19***	(0.06)	0.01	(0.06)	−0.04	(0.06)
Higher Diplomas	0.29***	(0.05)	0.18***	(0.05)	0.16**	(0.05)	0.02	(0.06)	−0.04	(0.05)
First Degree	0.61***	(0.06)	0.44***	(0.06)	0.38***	(0.05)	0.13*	(0.06)	0.10	(0.07)
Higher Degree	0.75***	(0.07)	0.53***	(0.07)	0.47***	(0.07)	0.16*	(0.07)	0.02	(0.06)
Household NS‐SEC (ref: Routine)										
Semi routine	0.04	(0.06)	0.03	(0.06)	0.04	(0.06)	0.02	(0.06)	0.02	(0.06)
Low sup and tech	0.02	(0.06)	−0.00	(0.06)	0.01	(0.06)	0.01	(0.06)	0.01	(0.06)
Small emp and s‐emp	0.02	(0.06)	0.00	(0.06)	0.01	(0.07)	0.01	(0.07)	−0.02	(0.06)
Intermediate	0.09	(0.06)	0.07	(0.06)	0.08	(0.06)	0.02	(0.06)	0.01	(0.07)
Lo manag/prof	0.11+	(0.06)	0.07	(0.06)	0.08	(0.06)	0.00	(0.06)	−0.02	(0.06)
Hi manag/prof	0.26**	(0.07)	0.18**	(0.07)	0.18*	(0.07)	0.06	(0.07)	0.02	(0.07)
Home owner (ref: do not own home)	0.01	(0.03)	0.02	(0.03)	0.02	(0.03)	0.02	(0.03)	0.01	(0.03)
Household income (log)	0.01	(0.02)	0.01	(0.02)	0.01	(0.02)	−0.02	(0.02)	−0.02	(0.02)
Mother's age	0.01**	(0.00)	0.01*	(0.00)	0.00+	(0.00)	0.00	(0.00)	0.00	(0.00)
Partner's age	0.00	(0.00)	0.00	(0.00)	0.00	(0.00)	−0.00	(0.00)	−0.00	(0.00)
Number of older siblings	−0.07***	(0.01)	−0.07***	(0.01)	−0.06***	(0.01)	−0.06***	(0.01)	−0.03**	(0.01)
Number of younger siblings	−0.02+	(0.01)	−0.03*	(0.01)	−0.03*	(0.01)	−0.03*	(0.01)	−0.02*	(0.01)
Speak other language at home (ref: English only)	0.08	(0.05)	0.10*	(0.05)	0.08+	(0.05)	0.18***	(0.05)	0.22***	(0.05)
Number of books at home (ref: <11)										
11–25			0.09*	(0.04)	0.08*	(0.04)	0.05	(0.04)	0.05	(0.04)
26–100			0.09**	(0.03)	0.08*	(0.03)	−0.00	(0.03)	−0.00	(0.03)
101–200			0.23***	(0.04)	0.19***	(0.04)	0.05	(0.04)	0.04	(0.04)
201–500			0.38***	(0.05)	0.32***	(0.05)	0.13**	(0.05)	0.11*	(0.04)
More than 500			0.59***	(0.07)	0.52***	(0.07)	0.29***	(0.06)	0.27***	(0.06)
Frequency reads to child (age 3) (ref: Never)										
Less often			0.12	(0.10)	0.11	(0.10)	0.09	(0.10)	0.05	(0.10)
Once or twice a month			0.06	(0.08)	0.05	(0.08)	0.01	(0.08)	−0.02	(0.08)
Once or twice a week			0.14*	(0.06)	0.12+	(0.06)	0.09	(0.06)	0.03	(0.06)
Several times a week			0.20**	(0.07)	0.16*	(0.07)	0.11+	(0.07)	0.06	(0.06)
Every day			0.24***	(0.06)	0.19**	(0.07)	0.14*	(0.06)	0.05	(0.06)
Reads for pleasure (age 11) (ref: Never)										
Less often					0.12*	(0.05)	0.11*	(0.05)	0.08+	(0.05)
At least once a month					0.14**	(0.05)	0.13**	(0.05)	0.09*	(0.04)
At least once a week					0.12**	(0.04)	0.10*	(0.04)	0.08+	(0.05)
Most days					0.39***	(0.04)	0.35***	(0.04)	0.30***	(0.04)
Plays musical instrument (ref: does not play)					0.10***	(0.02)	0.10***	(0.02)	0.07***	(0.02)
Main vocab score (*z*‐score)							0.19***	(0.02)	0.15***	(0.02)
Partner vocab score (*z*‐score)							0.14***	(0.02)	0.11***	(0.02)
Naming vocabulary *z*‐score (age 5)									0.20***	(0.01)
Picture similarities *z*‐score (age 5)									0.03**	(0.01)
Pattern construction *z*‐score (age 5)									0.09***	(0.01)
Constant	−2.80***	(0.48)	−2.98***	(0.48)	−3.19***	(0.48)	−2.47***	(0.45)	−0.86+	(0.45)
Adjusted *R* ^2^	11.33		13.38		15.32		18.02		22.05	

Note

Observations = 10,781.

Multiple imputation was applied to all missing data, including absent partners in single family households at MCS6.

*** *p* < .001;

** *p* < .01;

* *p* < .05;

^+^ *p* < .10.

Model 1 includes socioeconomic and demographic information and provides an indication of the magnitude of the associations between these variables and the child's vocabulary scores before any potential mediating factors have been accounted for. Parental education is strongly linked to the child's vocabulary. Having an undergraduate (bachelors) university degree or a higher (postgraduate) degree (compared to no qualifications) provides roughly three times the advantage associated with having a parent with a higher managerial or professional occupation (compared to a routine occupation) when both are included in the same model. Income and home ownership are not significantly associated with vocabulary, taking the other factors in the model into account.

Young people who are identified as ethnically Indian, Pakistani, black Caribbean, black African or “other” have lower scores than whites. There is no difference between boys and girls. As expected, young people with older siblings have a significant disadvantage in vocabulary scores, while the existence and number of younger siblings make little difference. The country of the United Kingdom is included, with London split from the rest of England, as educational policy and practice vary across these regions, and ethnic compositions vary widely. Living in Scotland or Wales is associated with a disadvantage, and London with an advantage, in vocabulary once individual characteristics are controlled. Both maternal age (in years) and child age (in months) are positively associated with the child's vocabulary score. The age range of the MCS births extended over a full calendar year. The coefficient associated with a month in age (0.01) can, therefore, usefully be compared to other coefficients in the model. For example, having a parent with an undergraduate degree is associated with five times the vocabulary advantage associated with one year in age and a parent with a postgraduate degree is associated with over six times the advantage of a year in age.

Model 2 introduces family cultural resources, in the form of books in the home and reading to the child at age three. Both of these variables are positive predictors of the young person's vocabulary. In this model, having over 500 books in the home is associated with a similar vocabulary advantage to having a parent with a postgraduate degree, and equates to around five times the advantage attributable to a year in age. The introduction of family cultural resources into the model mediates the parental education and social class effects to some extent.

Model 3 includes the child's own reading for pleasure and playing a musical instrument. Playing a musical instrument is associated with a positive difference in vocabulary equivalent to ten months in age. Reading for pleasure most days is associated with a differential over three times greater than the differential attributable to one year in age. However, these child activities do little to reduce the effects of parental education, social class, and cultural capital.

In model 4, we introduce the mother's and partner's vocabulary scores. Both, especially the mother's, are strongly independently associated with the child's score. Including parental vocabulary reduces the apparent influence of parental education, reducing the coefficients for a degree and higher degree by about half, and reducing lower levels of education to statistical insignificance. Social class also becomes statistically non‐significant in this model. The coefficients for the home literary climate are also substantially reduced, but the association with the child's own cultural activities is unaffected. While we treat parental vocabulary as a mediator of parental education, we acknowledge that a large portion of the gap in parental vocabulary is likely to be in place prior to parents gaining their highest educational qualification, and the fact that our measure of parental vocabulary is not time varying means that we cannot unpack the reciprocal relationship between parental vocabulary and educational attainment over time in this paper. Our interpretation of parental vocabulary as a mediator of parental education attainment simply means that part of the association of parental education on child vocabulary is explained by the higher vocabulary scores of more educated parents.

All ethnic differences become small and non‐significant in this model, with the exception that the Bangladeshi and Pakistani groups have a substantial advantage over whites once parental vocabulary is controlled. This suggests that Bangladeshi and Pakistani children have higher vocabulary scores than would be expected based on their parents’ scores, which are the lowest of any ethnic group.

Our final model includes the child's prior verbal and non‐verbal cognitive scores, naming vocabulary, picture similarities, and pattern construction at age five. As expected, these are strong predictors of attainment, particularly the vocabulary score. This model shows us which factors predict vocabulary at age 14 conditional on attainment at age five, bearing in mind that the age‐appropriate vocabulary test at age five of course differs from the vocabulary test at age 14. Parental vocabulary remains highly significant, whereas parental education and reading to the child at age three become non‐significant, their effect being fully captured by the child's cognitive scores at age five. The associations with books in the home and the child's own reading for pleasure are only slightly reduced, as these variables remain powerfully predictive of vocabulary at 14 conditioning on earlier cognitive scores.

As an indication of effect size, we converted the coefficients in this final model in terms of the raw test scores. Notable coefficients are as follows: a one standard deviation increase in verbal cognition at age five is associated with a 0.5 word increase in mean vocabulary scores (out of 20); a one standard deviation increase in maternal vocabulary is associated with an advantage of 0.4 words; a one standard deviation increase in partner's vocabulary equates to 0.3 words; more than 500 books in the home equates to 0.7 words; Bangladeshi ethnicity equates to 0.7 words; a non‐English language at home equates to 0.6 words, and reading for pleasure most days at age 11 equates to 0.8 words.

**FIGURE 1:**
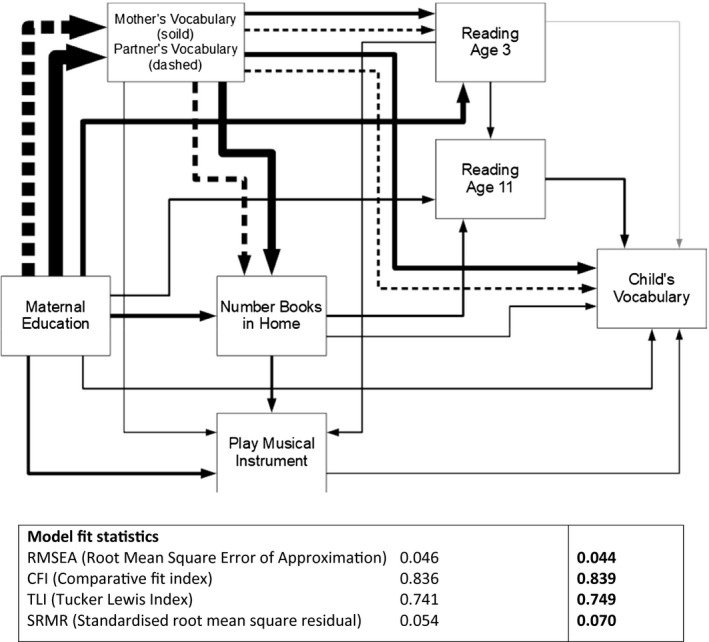
Path model: Simplified version of the regression model 4. Line widths proportionate to standardized coefficient values

In Table 4 and Figure 1, we provide a formal mediation analysis, based on a simplified path analysis version of the penultimate model (model 4), carried out in MPlus (Muthén & Muthén, 1998–2010), shown in Figure 1. This simplified model shows the relationship between parental education and offspring vocabulary, mediated by maternal and partner vocabulary and other factors Nearly a quarter (24%) of the relationship between parental education and child vocabulary is direct, with the remainder (76%) mediated by other factors in the model. The majority of this indirect effect (35% of the total effect) goes via maternal vocabulary, with an additional 20% via partner vocabulary. Maternal vocabulary is the most important mediator of parental education in the model. The effect of parental education is not fully mediated in this simplified model, whereas it is in the full regression model.

**TABLE 4:** Mediation of the relationship between parental education and offspring vocabulary via parental vocabulary

		Standardized coefficient	*SE*
	Total	0.291	0.015
	Total direct	0.069	0.017
	Total Indirect	0.222	0.014
Of which via	Mother vocabulary	0.101	0.010
Of which via	Partner vocabulary	0.059	0.009
	Other pathways	0.062	

## 6 CONCLUSIONS

Our central result is that parental vocabulary scores mediate a substantial share of the socioeconomic gradient in children's vocabulary at age 14. The importance of parental vocabulary is not surprising, but suggests that both the “cultural capital” and “home learning environment” literature have neglected a fundamental element of the learning resources that children have available at home—their parents’ own knowledge.

The paper advances the field of research into socioeconomic differentials in young people's language skills by providing evidence on the “word‐gap” based on large‐scale, nationally representative data on both parents’ and children's vocabulary scores. The raw inequalities that we find in parental vocabulary are startling. For example, parents with an undergraduate degree knew twice as many words on the assessment as parents with no qualifications. Though of course not directly comparable with Hart and Risley’s (1995) small‐scale study, which was carried out in another place and time, and using different methods, this difference is in line with the order of magnitude of the “word gap” found in Hart and Risley's work.

However, whereas Hart and Risley found similar social class differences in vocabulary for children as for their parents, we do not. The socioeconomic differentials that we found for young people at age 14 were marked, but substantially more modest than those found among their parents. Similarly, vocabulary gaps between ethnic groups were substantial in the parents’ generation, but slight for the children. This gives some grounds for optimism, in that socioeconomic differentials in vocabulary are not transmitted wholesale from parents to children. Children are exposed to vocabulary, not just from their parents, but from a range of sources including friends, teachers, books, TV, and the internet. Some of these wider exposures may mitigate the relationship between parental and child vocabulary. In particular, it is likely that schooling plays a role (Quinn, 2015a). However, it is of course possible that vocabulary gaps will widen substantially during the cohort members’ life course.

Our models of children's vocabulary at age 14 show that parental education appears to be a more consistent driver than other aspects of socioeconomic position of differentials in children's vocabulary scores. The differentials due to parental education were somewhat reduced by accounting for the home literary climate. In contrast, the child's own cultural activities, particularly reading, matter but do not mediate the differential due to parental education. This challenges the traditional cultural reproduction framework, to the extent that the child's own cultural participation appears to have little to do with the reproduction of socioeconomic differentials in attainment.

We have shown that parental vocabulary is a vital mediator of differentials in children's vocabulary according to parental education, and parental vocabulary also partly explains the apparent link between the home literary environment and children's vocabularies. This suggests that the omission of parental vocabulary from most previous models of children's language development, and indeed of their educational development more generally, may have led to a skewed and incomplete understanding of inequalities in children's outcomes, exaggerating the role of parental resources and behaviors which proxy parental language competencies (and may well also proxy other associated cognitive abilities). Furthermore, parental vocabulary strongly predicts language at 14, conditioning on cognitive scores at age five, suggesting that its influence is not restricted to early childhood development.

There is an extensive international literature on ethnic differentials in vocabulary and other cognitive test scores (Belsky et al., 2007), and our findings on the role of parental vocabulary in accounting for ethnic differentials in children's vocabulary provide a novel insight. We found that some groups of ethnic minority parents had substantially lower vocabulary scores than whites, and Pakistani and Bangladeshi parents had lower English vocabulary scores than their children. Ethnic gaps among the children's generation were smaller, but, controlling for socioeconomic and demographic factors, the Indian, Pakistani, black Caribbean, black African, and “other” ethnic groups remained at a disadvantage in their vocabulary scores compared to their white peers. The outstanding negative differentials in young people's vocabulary between some minority ethnic groups and whites were fully explained by differences in parental vocabulary. Our analysis also suggests that speaking a language other than English in the home is generally positive, once other factors are controlled (Marian & Shook, 2012; Portes & Rumbaut, 2001). There is great diversity between families with English as an additional language, which would demand an analysis primarily focused on this question to elaborate. Nevertheless, we can conclude that poor English language skills among parents present an obstacle for children, but this does not imply that the presence of an additional language in the home is detrimental in itself.

Despite the several strengths of our study, we acknowledge some limitations. First, attrition from the study since the baseline is just under 40%. We use Multiple Imputation to address this. Whilst it is difficult to know the extent to which there may be residual unobserved factors affecting attrition, in controlling for an extensive range of observables the issue is likely to be mitigated.

A second limitation is that we only have parental vocabulary measured at one time‐point, when the young person is aged 14, and for some parents, particularly those from immigrant groups, this may not accurately reflect their vocabulary earlier in the child's life.

A third limitation of this paper is that we are only able to report on the intergenerational transmission of vocabulary. A full assessment of the role of language in the process of “cultural reproduction” would require an assessment of later educational attainment and occupational outcomes. We intend to investigate these in future work.

A fourth limitation is that we are unable to address genetic heritability (Plomin & Deary, 2015). Evidence from the Dunedin cohort (Belsky et al., 2016) suggests that children born into socially disadvantaged families tend to have slightly below average polygenic scores for educational attainment and that these scores predict cognitive, educational, and socioeconomic attainment. This is beyond the scope of the current study, but future studies will be able to exploit the fact that the age 14 wave of MCS collected saliva from children and parents for subsequent DNA extraction.

From a theoretical point of view, our findings support the view that language skills are an important part of the resources that more privileged parents possess and are able, to some degree, to transmit to their children. This can be seen as supporting a Bourdieusian “cultural reproduction” perspective to a degree, yet this process is far from deterministic. We also find some support for Modood’s (2004) notion of “ethnic capital” overcoming a lack of traditional cultural capital, notably in the case of ethnically Bangladeshi and Pakistani children, whose English vocabulary scores are higher than would be expected given their parents’ low scores.

In policy terms, our findings should temper the tendency of some political commentators to blame socioeconomic differences in learning on deficits in working‐class parenting behaviors (Field, 2010; Telegraph, 2010). Our results suggest that children whose parents are less educated and those from particular ethnic minority groups may require additional input at school to support the development of a rich vocabulary, and encouraging independent reading is likely to be a useful tool in this regard, though we acknowledge that more research is needed to unpack the direction of causality between reading and cognitive and behavioral and emotional development. In the case of immigrant parents who lack English fluency, support for their English language development is likely to benefit their children, and this issue has increased in salience given the dramatic rise in the proportion of births to non‐UK women since the millennium.

Finally, our findings emphasize the value of including measures of parental cognitive skills, including language skills, in birth cohort studies, and other datasets, both as an important explanator and as a vital control variable. More research is needed internationally to examine whether the role of parental vocabulary varies across national contexts.

## Figures and Tables

**FIGURE A1:**
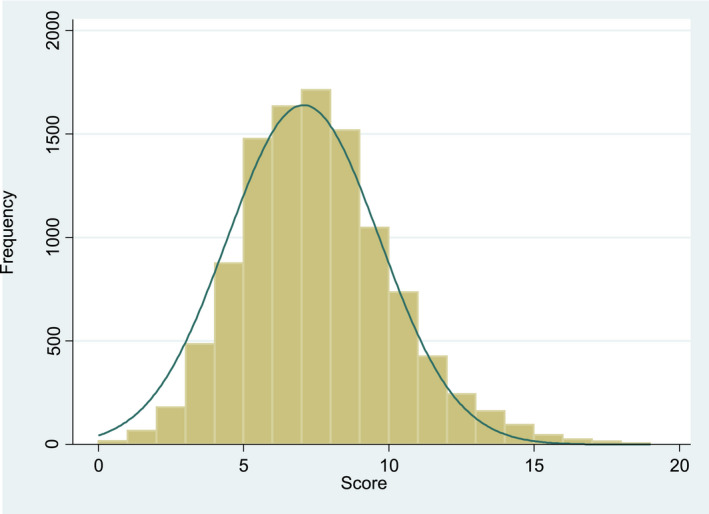
Young Person's vocabulary

**FIGURE A2:**
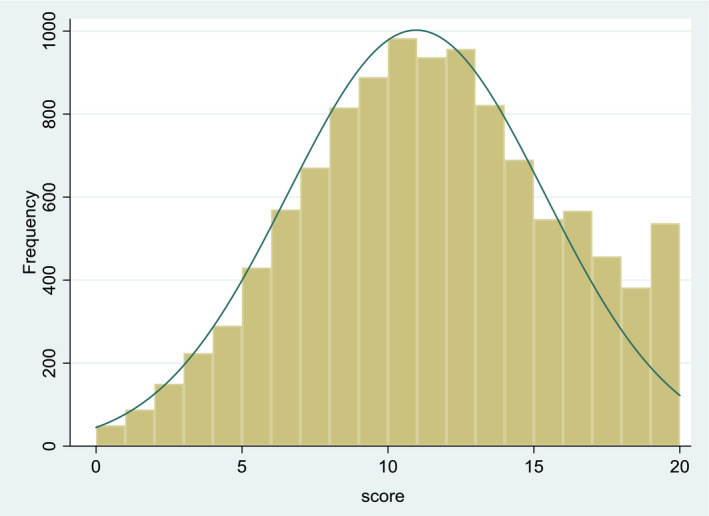
Mother's vocabulary

**FIGURE A3:**
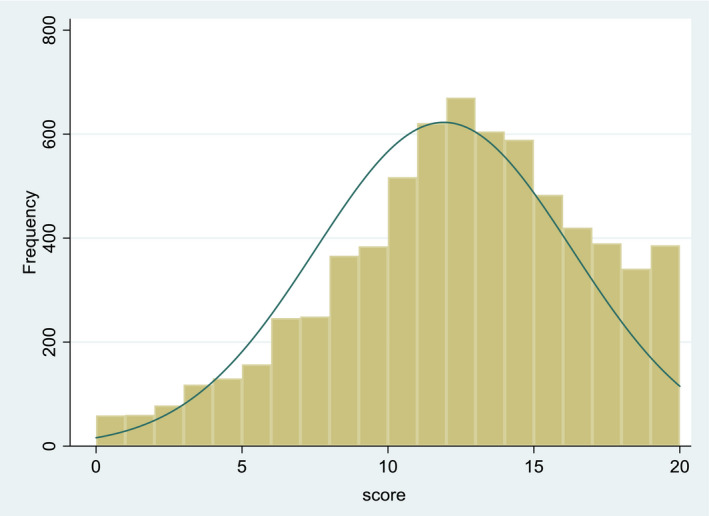
Father's vocabulary

## Data Availability

We are grateful to the Centre for Longitudinal Studies, UCL Institute of Education, for the use of these data and to the UK Data Archive and UK Data Service for making them available.

## Appendix

**TABLE A1:** Vocabulary scores internal reliability (20 item scale)

	Young person	Mother	Partner
Cronbach's alpha	0.543	0.839	0.851
KR20	0.522	0.839	0.852
Skewness	0.508	−0.019	−0.397
Kurtosis	3.601	2.442	2.744
